# Morphoscanner2.0: A new python module for analysis of molecular dynamics simulations

**DOI:** 10.1371/journal.pone.0284307

**Published:** 2023-04-27

**Authors:** Federico Fontana, Calogero Carlino, Ashish Malik, Fabrizio Gelain

**Affiliations:** 1 Center for Nanomedicine and Tissue Engineering (CNTE), A.S.S.T. Grande Ospedale Metropolitano Niguarda, Milan, Italy; 2 Fondazione IRCCS Casa Sollievo della Sofferenza, Unità di Ingegneria Tissutale, Foggia, Italy; Lawrence Livermore National Laboratory, UNITED STATES

## Abstract

Molecular dynamics simulations, at different scales, have been exploited for investigating complex mechanisms ruling biologically inspired systems. Nonetheless, with recent advances and unprecedented achievements, the analysis of molecular dynamics simulations requires customized workflows. In 2018, we developed Morphoscanner to retrieve structural relations within self-assembling peptide systems. In particular, we conceived Morphoscanner for tracking the emergence of *β*-structured domains in self-assembling peptide systems. Here, we introduce Morphoscanner2.0. Morphoscanner2.0 is an object-oriented library for structural and temporal analysis of atomistic and coarse-grained molecular dynamics (CG-MD) simulations written in Python. The library leverages MDAnalysis, PyTorch and NetworkX to perform the pattern recognition of secondary structure patterns, and interfaces with Pandas, Numpy and Matplotlib to make the results accessible to the user. We used Morphoscanner2.0 on both simulation trajectories and protein structures. Because of its dependencies on the MDAnalysis package, Morphoscanner2.0 can read several file formats generated by widely-used molecular simulation packages such as NAMD, Gromacs, OpenMM. Morphoscanner2.0 also includes a routine for tracking the alpha-helix domain formation.

## Introduction

Bio-molecular self-assembly has inspired the so-called “bottom-up” approach to designing self-assembling biomaterials [1–3]. In particular, many researchers focused their efforts in exploiting protein-protein interactions to fabricate new functional self-assembling biomaterials [4, 5]. Among self-assembling biomaterials, the self-assembling peptides (SAPs) hydrogels have found large applications in tissue engineering and regenerative medicine [6–9]. The limited control of structural, physical, and biochemical properties of SAPs hampers tailored applications in diverse fields. The reversible and temporary non-covalent interactions at the molecular level heavily affect the emergent properties of the SAPs at different scales [10, 11]. Molecular Dynamics (MD) simulations have proven to be a powerful tool for elucidating the complex molecular interplay at the basis of supramolecular biomaterials, unveiling protein-protein and protein-peptide interactions [12, 13]. Furthermore, MD simulations found applications in the study of intrinsically disordered proteins [14, 15]. Atomistic and coarse-grained (CG) molecular dynamics (MD) simulations have been used with empirical experiments to elucidate the self-assembly pathways and structuring propensities of several peptide sequences at the nanoscale level [16–18]. More in detail, molecular dynamics simulations showed that molten peptide oligomers could act as incubators for *β*-structuring [19–21]. Despite such achievements at the molecular level, it is still unclear how the oligomer-to-fibril transition emerges. Coarse-grained molecular dynamics (CG-MD), enabling the simulation of larger systems on longer simulation times, showed great potential for high throughput screenings of the self-assembling propensity of biomolecules [12, 16]. In addition, CG-MD simulations found applications for studying the self-assembling propensity and mechanical properties of different peptide sequences for a wide latitude of potential applications [12, 13, 17, 22]. Also, the knowledge of the time-dependent formation of secondary structures is crucial for a deeper understanding of the self-assembling phenomenon. Nonetheless, analytical tools for quantitative tracking secondary structure patterns (such as *β*-sheet and *α*-helix) over MD trajectories are still lacking. To this purpose we developed and validated Morphoscanner, a topological pattern recognition software, on diverse protein structures: [18], we used Morphoscanner to analyze MARTINI CG-MD and AA-MD simulations of peptide systems. Here we introduce Morphoscanner2.0, a new python library suitable for MD simulations of multimolecular systems. We strongly leverage graph theory in our software, since the clustering robustness is due to the graph representation of the molecular system, and represent a foundational part of the theoretical and implementation strategy of Morphoscanner [18]. We use NetworkX as a library to handle graphs combined with an optimized version of the depth-first search algorithm in our workflow [23]. The graph representation is accessible to the user and enables customized analysis. In particular, we have proven that Morphoscanner2.0 is suitable for tracking the emergence of secondary structure patterns and quantifying the transition entropy related to the self-assembly process. Morphoscanner2.0 makes available to the user the data to compute the transition entropy calculation associated with secondary structure transitions. In addition, we demonstrate the interoperability of Morphoscanner2.0 with different Python Libraries such as MDAnalysis, PyTorch, Scipy, and Matplotlib [24–30]. These features will open new strategies in the development of SAPs systems and other biomaterials. Indeed, Morphoscanner will provide the missing tools in the panorama of biomolecular simulations packages and is poised to change the current approaches for the analysis of molecular dynamics simulations, specifically of protein and peptides.

## Materials and methods

### Definition and use of native contact in Morphoscanner

For tracking molecular alignment, Morphoscanner calculates, leveraging Pytorch, and MDAnalysis [27, 29], the euclidean distance between the center of masses of each atom group pair that could represent amino acid residue or DNA base pairs, as shown in Eq (1) [18].

$$\begin{array}{c} D_{ij}=\sqrt{\sum_{i=1}^{3}\sum_{j=1}^{3}{\left(x_{i}-x_{j}\right)}^{2}} \end{array}$$ (1)

Then compute the system distance map that is eventually used for deriving the system contact map according to the definition in Eq (2).

$$\begin{array}{c} C_{ij}=\delta \left(D_{ij}-D_{0}\right) \end{array}$$ (2)

In Eq (2), *δ* is the Dirac measure, *D*_*ij*_ is the distance between center of masses of atom−groups i and j, *D*_0_ represents the threshold distance. For the identification of the *β*-sheet patterns, the definition of *C*_*ij*_ has been adapted as shown in Eq (3).

$$\begin{array}{c} \beta C_{ij}=\delta \left(D_{ij}-D_{0}\right) \end{array}$$ (3)

In this case, *D*_0_ stands for the distance between two *β*-strands according to the structural characterization of cross-*β* structures, like amyloid fibrils or peptide seeds. Usually *D*_0_ varies between 4.7 and 5.3 Å [31].

Instead, for the identification of the *α*-helix patterns, the definition of *C*_*ij*_ has been adapted as shown in Eq (4).

$$\begin{array}{c} \alpha C_{ij}=\delta \left(D_{ij}-D_{0}\right) \end{array}$$ (4)

In this case, *D*_0_ stands for the distance between two center of masses of two residues in a *α*-helix domain [32, 33]

As elucidated in the next sections, the structural feature assignment is not only base on the distance threshold but also on the pattern in the contact map.

### From the contact map to the reciprocal alignment among molecules

According to Eq (2), it is possible to derive contact maps to highlight different structural features by setting appropriate distance thresholds. Then, each element of the contact map can be assigned according to the Eq (5).

$$\begin{array}{c} CM_{ij}=C_{ij} \end{array}$$ (5)

For the identification of the the *β*-sheet patterns, the element of the contact map can be assigned as shown in Eq (6).

$$\begin{array}{c} CM_{ij}=\beta C_{ij} \end{array}$$ (6)

If the investigated molecular system consists of multiple molecules of equal length the reciprocal alignment can be represented using a square matrix, dubbed molecular contact matrix. Each element of this matrix is assigned according to the algorithm shown in the next paragraph.

### The kernel set generation

The first subroutine of Morphoscanner consists of generating a set of matrices, or kernels, for the molecular mutual arrangements. As shown in Algorithm 1, a kernel set is generated for describing the parallel and antiparallel shift arrangements between pairs of molecules.

**Algorithm 1** Morphoscanner procedure for building the reference matrices set

**procedure** Pattern Library(i,j,k)

 *L* ← ⌀     ▹ *L* is an 4 * *RES* long set of matrices, having dimension *RES*

 *RES* ← *length* *of* *polymer*

 **for** *k* **do**

  **for** *i* **do**

   **for** *j* **do**

    **if** *j*-*i*=*k* and *k* < *RES* **then**

     *L*_*i*,*j*,*k*_ = 1

    **end if**

    **if** *i*-*j*=*k* and *RES* < *k* < 2 * *RES* **then**

     *L*_*i*,*j*,*k*_ = 1

    **end if**

    **if** *i*+*j*=*n*+*k* and 2 * *RES* < *k* < 3 * *RES* **then**

     *L*_*i*,*j*,*k*_ = 1

    **end if**

    **if** *i*+*j*=*n*-*k* and 3 * *RES* < *k* < 4 * *RES* **then**

     *L*_*i*,*j*,*k*_ = 1

    **end if**

   **end for**

  **end for**

 **end for**

 **return** *L*

**end procedure**

Then, the parallel and antiparallel shift arrangements are described using the following matrix notation:

$$\begin{array}{c} P_{ij}^{+}=\delta_{i+k,j} \end{array}$$ (7)

$$\begin{array}{c} P_{ij}^{-}=\delta_{i-k,j} \end{array}$$ (8)

$$\begin{array}{c} A_{ij}^{+}=\delta_{n-i+k,j} \end{array}$$ (9)

$$\begin{array}{c} A_{ij}^{-}=\delta_{n-i-k,j} \end{array}$$ (10)

In the previous formulas *δ*_*ij*_ identify the Kronecker delta, n is the number of residues, *i*,*j* are the indexes of the residue (varying within *n*), and *k* is the shift value. This set of matrices describing the peptide interaction library can be represented using the following compact notation:

$$\begin{array}{c} L=L_{ijk} \end{array}$$ (11)

where *k* is the index of shift matrices in the library according to Algorithm 1.

### The molecular alignment matrix

Algorithm 2 describes the assignment of the element to the molecular alignment matrix. Each element of the molecular alignment matrix correspond to the kernel, in position *K*, that maximize the normalized cross-correlation (NCC) with the area of the contact matrix, corresponding to the interaction between two molecules [29, 34, 35].

$$\begin{array}{c} NCC\left(p,q,k\right)=\frac{\sum_{i=0}^{RES-1}\sum_{j=0}^{RES-1}BB\left(i+p*RES\right)\left(j+q*RES\right)*L\left(i,j,k\right)}{\sqrt{\sum_{i=0}^{RES-1}\sum_{j=0}^{RES-1}BB\left(i+p*RES\right)\left(j+q*RES\right)}\sqrt{\sum_{i=0}^{RES-1}\sum_{j=0}^{RES-1}L\left(i,j,k\right)}} \end{array}$$ (12)

**Algorithm 2** Morphoscanner procedure for retrieving the molecular interaction matrix

**procedure** Molecular Alignment

 *P* ← ⌀ ▹ P is a matrix of size *nmol* x *nmol*, that is the number of molecules in the multimolecular system

 **for** *p* **do**

  **for** *q* **do**

   **if** *NCC*(*p*, *q*, *K*) ⇒ *max*(*NCC*(*p*, *q*, *k*)) **then**

    *P*_*p*,*q*_ = *K*        ▹ See Eq 12

   **else**

    *P*_*p*,*q*_ = 0

   **end if**

  **end for**

 **end for**

 **return** *P*

**end procedure**

### Protein structural domains

The identification of structural domains of proteins can be performed by recurring to the analysis of the contact map (See Eq 5 for details).Different tools are available for retrieving protein contact maps. The possibility of performing this analysis over time and with manageable graphical tools is the main contribution of Morphoscanner2.0 to this field.

#### *α*-helix and *β*-turn identification

Morphoscanner2.0 contains a routine for investigating the dynamics of the *α*-helix and *β*-turn domains. This routine is shown in Algorithm 3, and its implementation in S4 and S5 Figs and Fig 4. Such procedure relies on the identification of *α*-helix contact, according to the Eq 2 with *D*_0_ in the range from 5.1 to 6.3 Å [32, 33]. More in detail, the algorithm identifies a couple of non consecutive residues, whose distance of center of masses is lower than threshold distance,*D*_0_. Then, the algorithm checks if the residues are separated by more than 3 and less than 6 residues. In this case, the algorithm identifies an *α*-helix. Otherwise, it identifies a *β*-turn. It calculates number of residues in the *α*-helix, *β*-turn, the pace of the helix and the length of *β*-turn. In addition, it returns the number of protein segments that correspond to *α*-helix and *β*-turn pattern.

**Algorithm 3** Morphoscanner procedure for identifying *α*-helix and *β*-turn patterns

**procedure** Identification *α*-helix Domains(CM)

 *Countα* ← ⌀

 *Countβ* ← ⌀

 *Pace-helix* ← ⌀

 *Lengthβ* ← ⌀

 *α* − *helix* ← ⌀

 *β* − *turn* ← ⌀

 **for** i **do**

  **for** *j* ≥ *i* + 1 **do**

   **if** *CM*_*ij*_ == *αC*_*ij*_ **then**

    **if** *j* − *i* ∈ (3, 6) **then**

     *Countα* ← (*Countα*+1)

     *Pace* − *helix* ← (((j− i)*(*Count*_*α*_ − 1) + *Pace* − *helix*)/*Count*_*α*_)

     *α* − *helix*[*Countα*] ← *j*

    **else if** *j* − *i* ∉ (3, 6) **then**

     *Countβ* ← (*Countβ*+1)

     *Lengthβ* ← (((j − i)*(*Count*_*β*_ − 1) + *Lengthβ*)/*Count*_*β*_)

     *β* − *turn*[*Countβ*] ← *j*

     **return** Pace-helix, Count-*α*, *α*-helix, Count-*β*, Length*β*, *β*-turn

#### *β*-sheet identification

This Morphoscanner subroutine, for the identification of *β*-sheet structures, uses the *β*-contact map (See Eq 6 and Algorithm 4) and the strand interaction matrices, P. In detail, the algorithm identifies a potential triplet of strands making a putative *β*-structure in the matrices P [18].

Then using the information of the position of the triplet, it considers the area of the system backbone contact matrix, corresponding to the interaction between the first pair of strands, and reduces this area to a row vector, as shown below:

$$\begin{array}{c} v_{r}=\left(\sum_{i=0}^{n}\left(A\left(i,1\right)\right),\sum_{i=0}^{n}\left(A\left(i,2\right)\right),\ldots ,\sum_{i=0}^{n}\left(A\left(i,n\right)\right)\right) \end{array}$$ (13)

Morphoscanner identifies the area corresponding to the other pair of strands, giving a column vector

$$\begin{array}{c} v_{c}=\left(\sum_{j=0}^{n}\left(A\left(1,j\right)\right),\sum_{j=0}^{n}\left(A\left(2,j\right)\right),\ldots ,\sum_{j=0}^{n}\left(A\left(n,j\right)\right)\right) \end{array}$$ (14)

Finally, the projection of *v*_*r*_ on *v*_*c*_ is calculated as a dot product

$$\begin{array}{c} v_{p}=v_{r}*v_{c} \end{array}$$ (15)

The number of consecutive residues defining a structuring *β*-sheet along the covalent bonds direction is calculated as the maximum number of elements included between two non-null elements. In this way, *β*-sheet structures are identified as curved rectangular 2D-lattices whose dimensions are defined by strands and by the number of backbone grains [18].

**Algorithm 4** Morphoscanner procedure for identifying *β*-sheet domains

**procedure** Identification Structural Domains(P,CM)

 **for** p **do**

  **for** q > p **do**

   **if** *P*_*p*,*q*_ ≠ 0 **then**

    *v*_*r*_ ← *v*_*r*_(*CM*, *p*, *q*)      ▹ See Eq 13

    **for** > *q* **do**

     **if** *P*_*q*,*s*_ ≠ 0 **then**

      *v*_*c*_ ← v_*c*_(*CM*, *p*, *q*)      ▹ See Eq 14

      **if** *v*_*c*_ ⋅ *v*_*r*_ ≠ 0 **then**

       **return** p,q,s

      **else**

       **return** p,q

### Usage of Morphoscanner

In this section, the core workflow and basic plotting functions of Morphoscanner are being introduced. As shown in S1 Fig, the class **morphoscanner.trajectory.trajectory()** instantiates the class instance **trajectory()**, that is responsible to perform the analysis on a MD trajectory. **trajectory()** parses the data using **MDAnalysis.Universe()** parser. MDAnalysis is able to read multiple file format, from multiple MD software, and is up-to-date with the latest file format [25].

The instance **trajectory()** performs the analysis, saves the results of the analysis, and has the capability to visualize the computed data, as shown in S2 Fig.

The trajectory() class need few arguments to be instantiated and start the analysis:

1. **_sys_config** that is the file path of the initial configuration of the system
2. **_sys_traj** that is the path of the trajectory file.
3. The parameter **select** indicates the atoms that will be used to perform the analysis. Atoms definitions are taken from the MARTINI 2.2 [36]

The default selection selects all the amino acids backbone beads, labeled with *BB* in MARTINI 2.2 [36].

The available attributes for the instantiated object are **number_of_frames** and **universe** [25].

The exploratory step, **explore()**, is necessary to initialize a trajectory. If successful, this step returns information about the number of frames in trajectory, the number of proteins and their length.

The MD trajectory can be analyzed by considering different sampling intervals, using the **compose_database()** method.

After the frames sampling and data retrieval, each sampled frame of the system is analyzed with our algorithms, to search for the emergence of complex structures that match *β*-sheet patterns, according to the Algorithm 4. The dedicated method is **analyze_inLoop()**, which takes different parameters such the threshold distance (**threshold**) for defining contact (See also Eqs 1 and 3 for the geometrical definition). In addition, this method takes the number of contacts between two peptides to consider them as forming *β*-sheet structures (**minimum_contact**). The method **analyze_InLoop()** can be parallelized on CPU or GPU. The **helix_score()** method is used to track the *α*-helix domain dynamics. This method iterates the Algorithm 3 on each frame of the MD simulation. The method **get_data()** run a set of other methods that recover data from the analysis. The obtained data are saved in a pandas dataframe.

The visualization of the results relies on an ensemble of methods, as shown in S2 Fig. The method **plot_graph()** represents a graph that quantify the interactions between different protein or peptides, shown in S4 and S6 Figs. In particular, this method plots the graph of one of the sampled frames with qualitative visual indications of the type of interactions among different proteins or peptides. The method **plot_frame_aggregates()** is used for plotting a frame with a color code that identify the sense of the majority of contacts in a cluster, as shown in Fig 4B. The method **plot_contacts()** plots the ratio between antiparallel and total contact for each sampled frames, as shown in Fig 4C. This information can be used for assessing the overall alignment of peptides within *β*-sheet structures. In addition, this metric can be used to validate the SAPs MD simulations by comparing it to the ATR-FTIR analysis of SAPs hydrogels [37]. The method **plot_peptides_in_beta()** plots the ratio between the number of peptides that form *β*-sheet structures and the total number of peptides, identified through the Algorithm 4, as shown in Fig 4D. The methods **plot3d_parallel()**, **plot3d_antiparallel_negative()**, **plot3d_antiparallel_positive()** and **gnuplot_shift_profile()** return the distribution of shift values over time, as shown in Fig 5A–5C.

## Results and discussion

Previously, we proved that Morphoscanner recognizes the *β*-sheet structures in both atomistic and CG protein structures, providing additional information about the relative orientation and alignment of *β*-strands within *β*-sheet domains. Furthermore, we included Morphoscanner in a new high-throughput workflow to investigate different facets of self-assembly [18]. Thanks to its compatibility with the MARTINI CG-MD simulations approach, we elucidated the self-assembly process of BMHP1-derived SAPs, (LDLK)_3_ SAPs, and CAPs [8, 9]. Such workflow unveiled the aspects behind the thermodynamic stability of peptide aggregates. On the one hand, BMHP1-derived SAPs formed parallel *β*-sheet that might hamper a further evolution of the molten particles. On the other hand, (LDLK)_3_ and CAPs formed anti-parallel *β*-rich aggregates that might evolve toward cross-*β* packing [18]. Additional applications of Morphoscanner consisted of the analysis of steered MD simulations. We proposed and validated an innovative fine-to-coarse molecular modeling approach to elucidate the structure–mechanics relationship of peptide systems at the nano- and micro-scales. We investigated SAPs structuring and mechanical features through atomistic and GoMARTINI-based MD simulations [22, 38, 39]. The analysis with Morphoscanner of these simulations provided insights into the length-dependent failure mechanism of SAP fibrils. Then, Morphoscanner laid the foundation for reliable mesoscale models [22]. In this work, we introduce the latest new version of Morphoscanner and its interoperability with different Python libraries [24–26] In particular, we focused our efforts in the development of an *α*-helix tracking module [40]. We made this choice because *α*-helix structural domains play a pivotal role in DNA-protein interactions and in prion disease [41–44]. In addition, such implementation will allow tracking conformational transitions by using a quantitative methodology in MD simulations [45]. Then, we discuss the validation of the *α*-helix tracking module implemented in Morphoscanner2.0 and its applications to MARTINI CG-MD simulations of peptide systems [36].

### Analysis and visualization of *α*-helix structural domains

**Fig 1:**
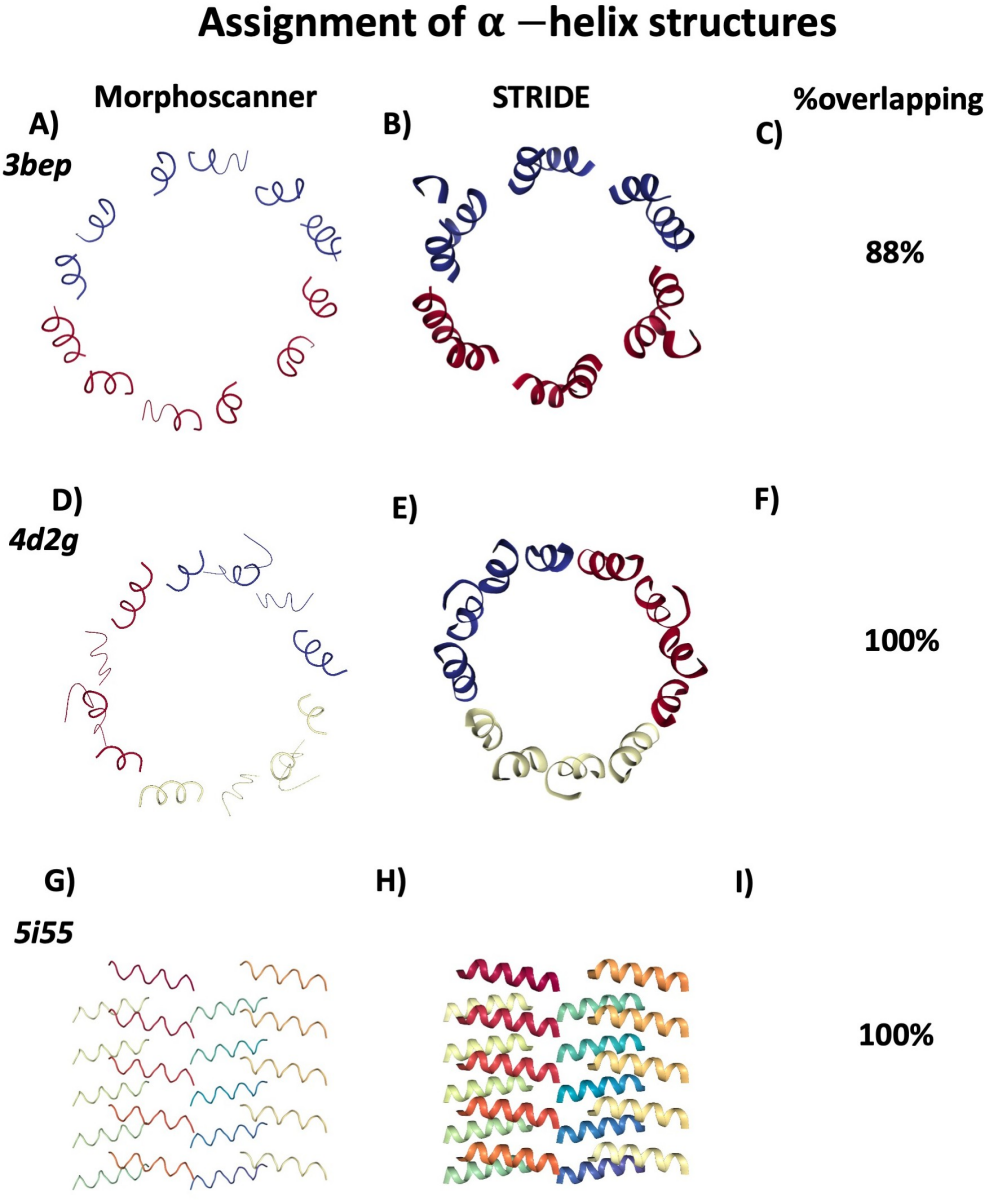
Validation of *α*-helix module of Morphoscanner on different protein structures. A series of PDB structures were analyzed with STRIDE web server and Morphoscanner2.0. In the first column, CG structures are visualized highlighting *α*-helix identified through Morphoscanner2.0. In the second column, atomistic structures are visualized highlighting *α*-helix identified through STRIDE. The overlapping percentages calculated by Stride and Morphoscanner (MS) are shown in the third column. The Morphoscanner2.0 analyses of 3bep, shown in A) are in agreement with STRIDE analyses. Indeed, as shown in *A*) and *B*) Morphoscanner2.0 correctly identifies all the *α*-helix domains. Similar results from the analysis of 4d2g and 5i55 (See D,E,F and G,H,I).

In our previous work, we validated Morphoscanner using a set of PDB structures eventually mapped according to MARTINI CG force field [18]. Similarly to what has be done for the *β*-structures recognition module, we have validated the Algorithm 3 considering the set of PDB structures shown in Fig 1 (See also S4 Fig for the command lines). Thanks to web server STRIDE [46], the secondary structures assignment for each PDB structure could be readily computed (S3 Fig for details about the validation protocol). The percentage of *α*-helix content has been calculated and compared with the corresponding value from Morphoscanner (See Fig 1 *C*, *F*, *I* and S3 Fig for more details). In addition, as shown in S6 Fig, Morphoscanner return the graphical representation of the alignment among the different molecular sub-units. More in details, 3bep the *Escherichia coli* *β* clamp is a subunit of the DNA polymerase III holoenzime which consists of two identical subunits, made of 366 residues each one. These subunits are antiparallel aligned as highlighted in S6 Fig *A* [47]. Instead, 4d2g the DNA-sliding clamp proliferating cell nuclear antigen (PCNA) is a homotrimeric ring that encircles DNA and and anchors binding partners, preventing them from falling off the genomic template. The three subunits of the PCNA sliding-clamp are antiparallel aligned as shwown in S6 Fig *B* [48]. The PSMα3 (5i55 in Fig 1) is a virulent 22-residue amyloid peptide secreted by *Staphylococcus aureus* that consists of 20 different *α*-helix peptides organized as a cross-*α* structures. As shown in S6 Fig *C*, the *α*-helix peptides are parallel aligned, whereas the *α*-sheets are antiparallel aligned [49]. So, the Morphoscanner analyses of 3bep, 4d2g and 5i55 were in agreement with STRIDE analysis. Indeed, the visualization of the *α*-helix domains of 3bep, shown in Fig 1A and 1B, confirmed the accuracy of the analysis. Similar conclusion can be drawn for 4d2g and 5i55, as shown in Fig 1D, 1E, 1G and 1H respectively.

### Tracking of secondary structures of *α*-helix forming peptides in CG-MD simulations

Starting from the results of the work of Ho and Dill [50], we selected 14 peptide sequences characterized by a high propensity of folding in *α*-helix. Then, we analyzed the *α*-helix and *β*-sheet folding propensity by combining MARTINI CG-MD simulations and Morphoscanner2.0 [36, 51].

Algorithm 5 iterates on each frame of the CG-MD simulation. As shown in the S5 Fig, Morphoscanner quickly identifies the protein conformations with the highest *α*-helix or *β*-turn content, as summarized in the Tables 1 and 2 respectively. In addition, Morphoscanner computes the average structuring propensity and stability. The stability of the structural domain folding considers the number of frames that correspond to a folding content higher than the average (*N*_*frames*_(*Fold*)), as shown in Eq 16 (See S4 Fig for the related Python scripts).

$$\begin{array}{c} Stability=N_{frames}\left(Fold\right)/N_{frames}*100 \end{array}$$ (16)

**Table 1:** Analysis of MARTINI CG-MD simulations of *α*-helix forming peptides. *Max*(*α*−*helix*) refers to the maximum *α*-helix content for the designated peptide sequence. *Frames*_*Max*(*α*−*helix*)_ is the count of frames that correspond to the maximum *α*-helix content. <*α*-helix> is the average *α*-helix content computed along all the CG-MD trajectory. The definition of Stability is shown in Eq 16.

ID	Sequence	Max (*α*-helix)	Frames(Max (*α*-helix))	<*α*-helix>	Stability
M11	IRLFKSHP	50	18	13.84	30.76
M13	HPETLEKF	62.5	14	22.75	58.24
M14	TLEKFDRF	62.5	1	17.25	43.05
M17	HLKTEAEM	62.5	5	20.41	52.44
M21	DLKKAGVT	62.5	1	16.13	40.95
M34	IPIKYLEF	62.5	10	18.06	45.35
M37	SEAIIHVL	62.5	1	17.5	45.5
M38	IIHVLHSR	62.5	56	31.2	52.14
M39	VLHSRHPG	62.5	7	25.43	32.46
M40	SRHPGNFG	62.5	6	27.05	41.85
M46	LELFRKDI	50	20	11.71	58.24
M47	FRKDIAAK	50	4	8.65	48.25
M48	DIAAKYKE	50	7	11	54

**Table 2:** Transition entropy from *α*-helix to *β*-turn structure. *Max*(*β*−*turn*) refers to the maximum *β*-turn content for the designated peptide sequence. *Frames*_*Max*(*β*−*turn*)_ is the number of frames that correspond to the maximum *β*-turn content. <*β*-turn> is the average *β*-turn content computed along all the CG-MD trajectory. The definition of Stability is shown in Eq 16 Relative entropy measures the the amount of information gained in switching from *α*-helix to *β*-turn structures [52].

ID	Max (*β*-turn)	Frames(Max (*β*-turn))	<*β*−*turn*>	Stability	Transition Entropy
M11	25.0	19	1.74	12.08	1.04
M13	37.5	6	3.40	22.87	1.52
M14	37.5	9	5.29	33.96	0.68
M17	37.5	6	3.90	25.77	1.12
M21	37.5	7	3.18	21.07	0.82
M34	37.5	3	2.23	15.28	1.26
M37	37.5	3	3.15	20.67	1.09
M38	37.5	4	3.18	21.07	2.63
M39	37.5	4	4.10	27.57	2.58
M40	37.5	8	5.20	33.16	1.84
M46	37.5	4	1.91	13.48	0.67
M47	37.5	1	1.17	8.09	0.56
M48	25.0	9	1.08	7.79	0.77

The analysis with Morphoscanner of 10-ns long MARTINI CG-MD of *α*-helix forming peptides(See Table 1) classified emerging structural domains in terms of extension and stability.

#### Alpha-helix propensity of peptide derived from the myoglobin

**Fig 2:**
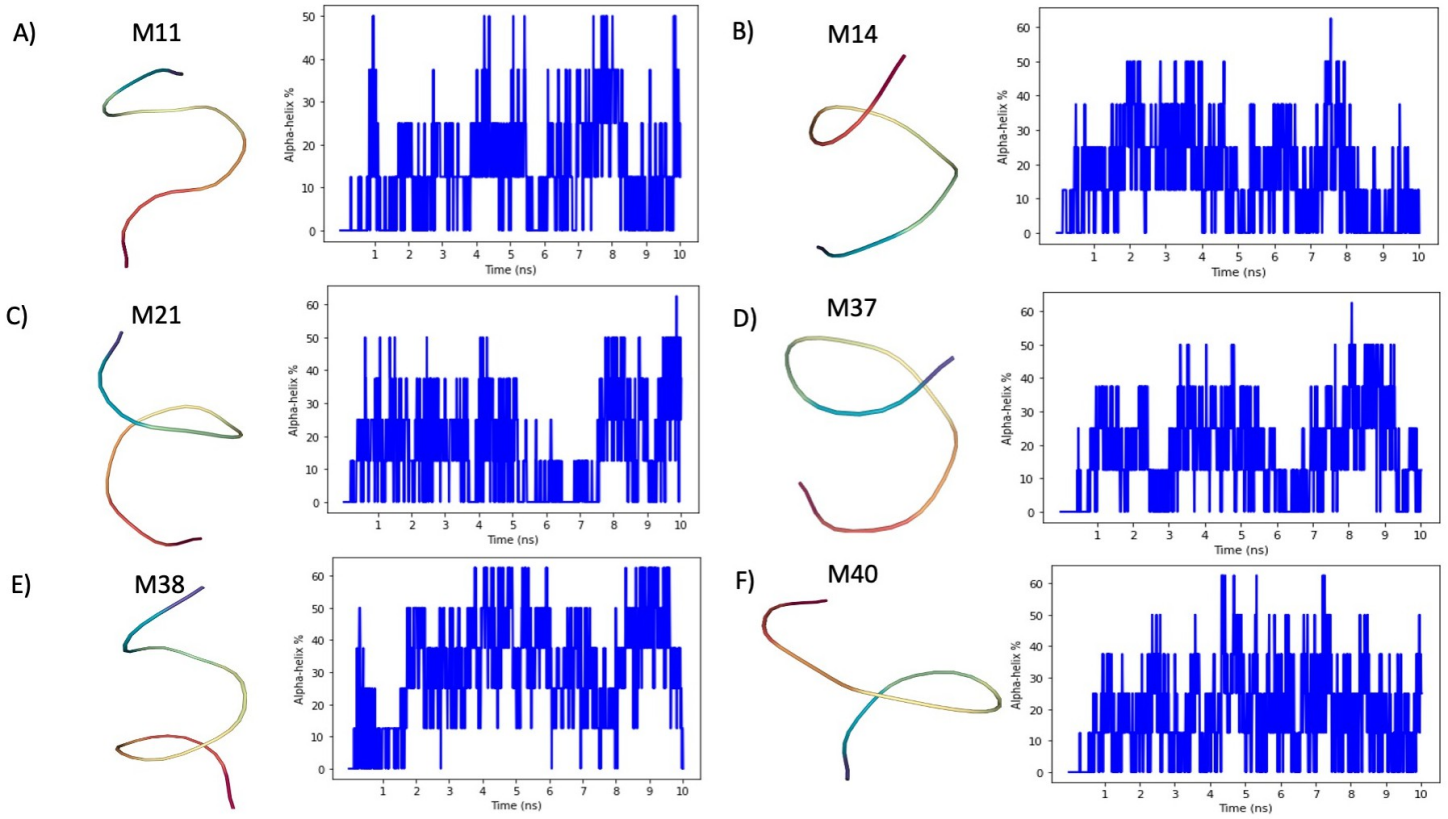
Tracking of *α*-helix structures in CG-MD simulations. A) The peptide M11 is characterized by a low *α*-helix structuring propensity. Indeed, Morphoscanner returns an average content of 13.84% *α*-helix structures and a peak at 50%. In addition, the stability scores is lower than 31%. B) Peptide M14 is characterized by a good propensity to fold in *α*-helix, indeed the average *α*-helix content is equal to 22.75% with a peak at 62.5%. C) Peptide M21 shows a similar behavior to that observed for Peptide M14. D) Peptide M37 tends to fold in *α*-helix analogously to Peptide M21 and M14. E) Peptide M38 is the peptide with the highest propensity to fold in *α*-helix. Indeed, the average *α*-helix content is 31% and the stability score is equal to 52.14%. F) Peptide M40 shares similar pattern to peptide M11, but its tendency to fold in *α*-helix is higher. Indeed, the stability score is equal to 41.85%.

**Fig 3:**
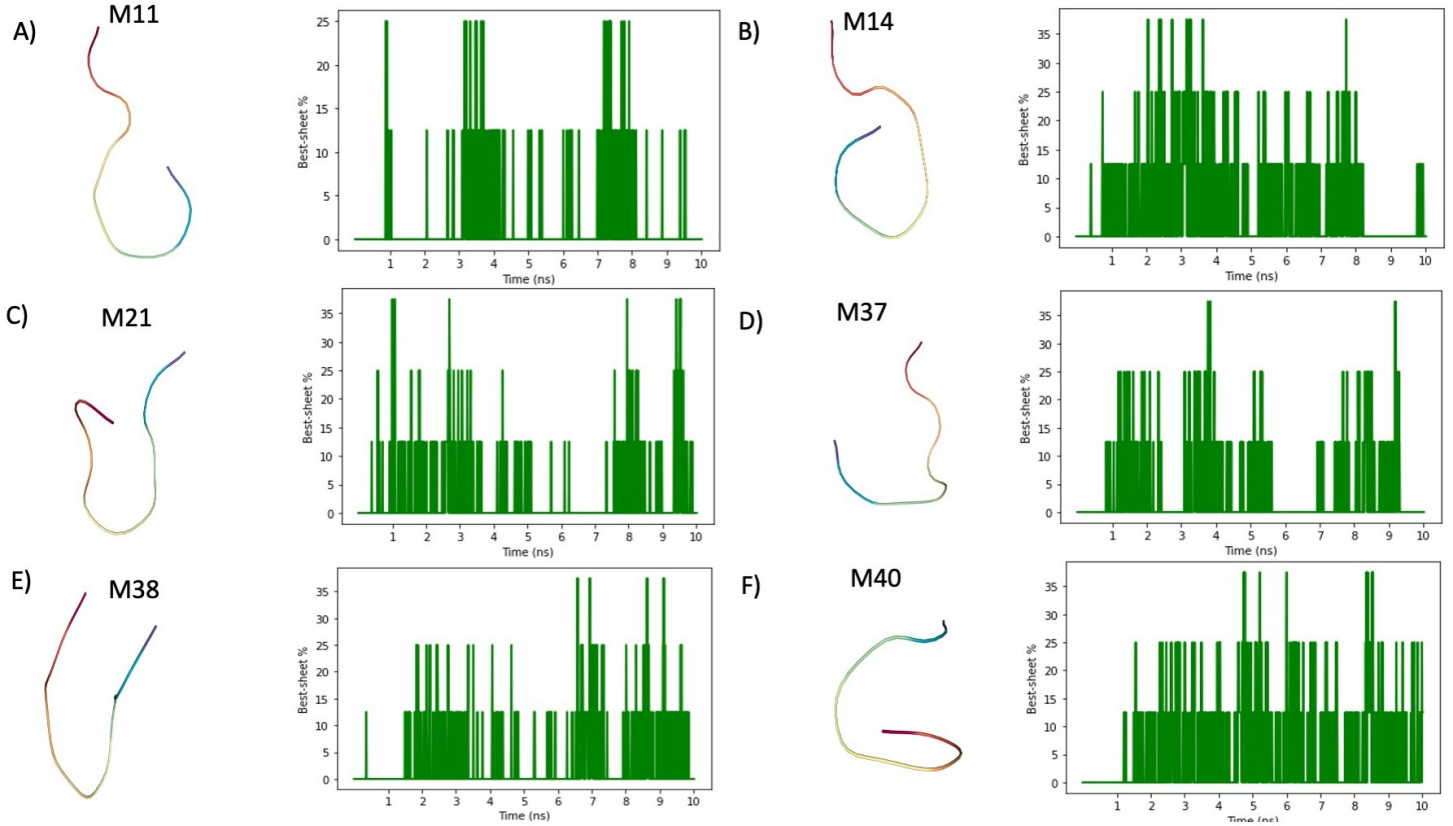
Tracking of *β*-turn structures in CG-MD simulations. A) The peptide M11 is characterized by a low *β*-turn structuring propensity. Indeed, Morphoscanner returns an average content of 1.74% *β*-turn structures and a peak at 25%. In addition, the stability scores is 12.08%. B) Peptide M14 is characterized by a low propensity to fold in *β*-turn, indeed the average *β*-turn content is equal to 5.29% with a peak at 37.5%. C) Peptide M21 shows a similar behavior to that observed for Peptide M14. D) Peptide M37 tends to fold in *β*-turn analogously to Peptide M21 and M14. E) Peptide M38 shows a similar behavior to peptide M37. Indeed, the average *β*-turn content is 3.18% and the stability score is equal to 21.07%. F) Peptide M40 shares a similar pattern to peptide M11, but its tendency to fold in *β*-turn is higher. Indeed, the stability score is equal to 33.16%.

The analysis with Morphoscanner2.0 of this set of simulations gave information about the structural stability of each *α*-helix forming peptide. To quantify the structural transition entropy, we calculated the relative entropy (See Table 2 and S5 Fig), also known as Kullback-Leibler divergence, by considering the time series in Figs 2 and 3, S7 and S8 Figs [52], as shown in Eq 17

$$\begin{array}{c} RE_{\alpha ,\beta }=\sum_{t}\beta_{turn}\left(t\right){\text{log}}_{2}\left(\beta_{turn}\left(t\right)/\alpha_{helix}\left(t\right)\right) \end{array}$$ (17)

Higher values of relative entropy point out to less probable structural transitions. According to the transition entropy shown in Table 2, peptides M13, M34, M38, M39, and M40 exhibit a higher *α*-helix folding propensity.This feature could be related to two different patterns in the sequence set consisting of the M13 and M34 peptides and in the sequence set of the M38, M39, and M40 peptides. Indeed, peptides M13 and M34 features charged amino-acid like Lysine, Glutamic Acid and Histidine. Instead, peptides M38, M39, M40 mainly consist of hydrophobic apolar amino-acids like Valine, Serine, Glycine, Isoleucine.

### Unveiling the triggering mechanism that leads to peptide self-assembly with Morphoscanner2.0

**Fig 4:**
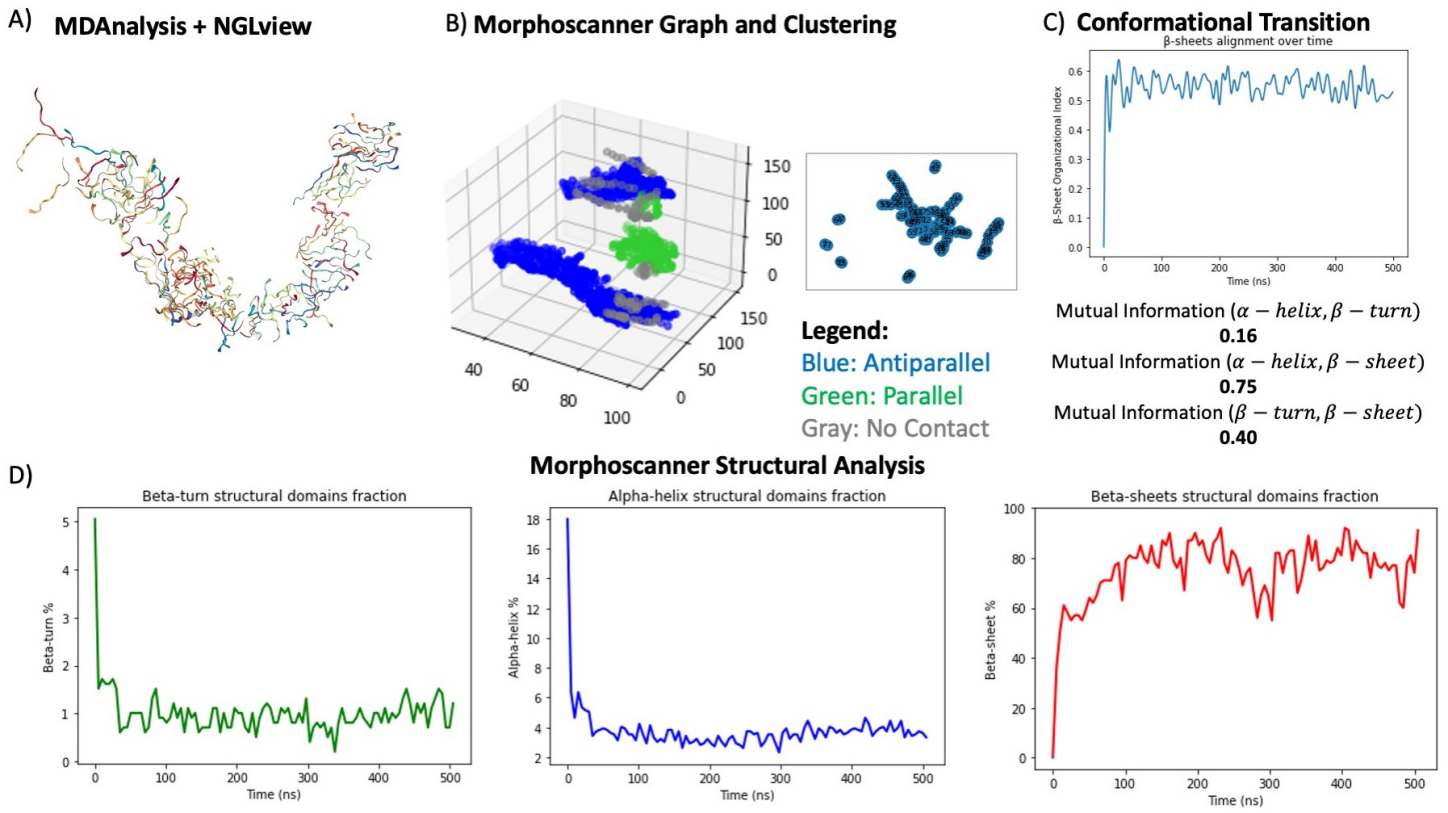
Analysis of atomistic MD simulation of SAPs B24. As shown in A) Morphoscanner can be used, in combination with NGLview, for obtaining graphical representation of the snapshot of MD trajectories similarly to VMD. B) By considering a single frame Morphoscanner returns the graph and clustering, as highlighted in the insert, of the peptides in the MD simulations. C) The conformational transitional analysis points out that the formation of antiparallel *β*-sheets domains relies on the structural transitions of *α*-helix peptides. See S5 Fig for details about the calculation of mutual information. Indeed, as shown in D), at the beginning of the simulations 18% of the peptides are folded in *α*-helix like structures, whereas just 5% of the peptides adopts a *β*-turn conformation. Then, the analysis reveals that the B24 peptides progressively form *β*-sheet rich aggregates from a mixture of *α*-helix and *β*-turn conformers.

**Fig 5:**
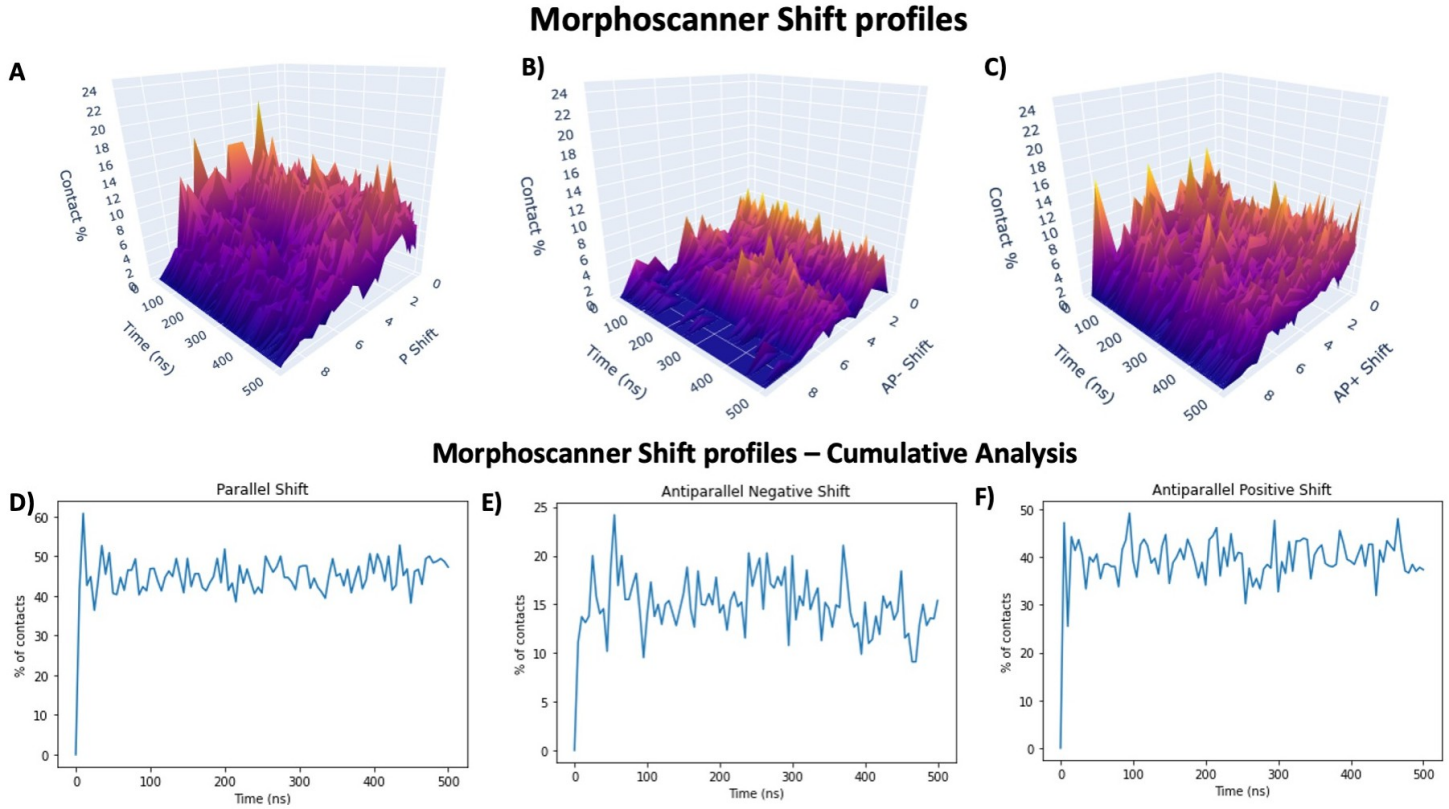
Shift profiles of MD simulations of B24 SAPs. According to Fig 4, B24 mainly form *β*-sheet rich aggregates where peptides are mainly antiparallel aligned. Indeed as shown in A), B) and C), B24 SAPs mainly form antiparallel *β*-sheet structures (40% of the contacts are classified as AP+, 15% of contacts are classified as AP-). The same statistics are also recapitulated in D), E) and F. In this way, it is possible to further rationalize the analysis performed in Fig 4C.

We used Morphoscanner combined with long and large-scale restrained atomistic molecular dynamics (MD) simulations for elucidating the self-assembling and pattern organization of the B24 SAPs. Due to the high speed and compatibility of Morphoscanner2.0 with different operating systems, the analysis have been performed using a workstation. More in details, the MD simulations were steered using ssNMR secondary structure information via TALOS+. We simulated the self-organization of one hundred B24 peptides that were initially randomly distributed in a water-filled box [53, 54]. As shown in Fig 4 and in according to our previous works, B24 peptides rapidly formed smaller *β*-sheet rich clusters within the first 50 ns [18, 53]. Due to the preference for *β*-strand conformation and their amphiphilic nature, the peptides mostly assemble into elongated clusters. The visualization with the NGLview widget (See Fig 4A) confirmed the previous consideration about the overall shape of the elongated fiber-like construct reminiscent of the flat B24 fibers previously observed by AFM. Thanks to the analysis with Morphoscanner, we found that SAPs within clusters are mainly antiparallel aligned as shown in Figs 4B and 5A–5C). In particular, the shift profiles in Fig 5 gave quantified the *β*-strands displacements over time within *β*-sheets aggregates, according to parallel (Fig 5A), negative antiparallel (Fig 5B) and positive antiparallel (Fig 5C) alignment. (See Eqs 7, 8, 9 and 10 for details). These considerations are also corroborated by the analysis of the *β*-sheet organizational index [37, 55]. The organizational index is the ratio between anti-parallel and parallel organization of the *β*-strands. In this simulation, the average value of the *β*-sheet organizational index is higher than 0.5 (See Figs 4C and 5D–5F). As shown in Fig 4D by comparing the *β*-turn, *α*-helix and *β*-sheet domain fractions over time, we gained additional insights about the trigger mechanism that lead to the formation of *β*-sheet-based clusters of SAP B24. Indeed, at the beginning of the simulations 18% of the peptides adopt an *α*-helix conformation, whereas 5% of the B24 peptides adopt a *β*-turn conformation. To measure the intrinsic dependence between structural domains, we calculated mutual information by analyzing the output of Morphoscanner, shown in Fig 4D, with dedicated functions from the PyInform library, as shown in S5A–S5D Fig [56]. The measurement of mutual information between *α*-helix and *β*-sheets structural domains fractions unveil an high dependency (Mutual information *α*-helix and *β*-sheets is equal to 0.75, See Fig 4C). Similar considerations can be asserted with the measure of the mutual information of *β*-turn and *β*-sheets structural domain fractions (See Fig 4C). In this case, the value of the mutual information is equal to 0.4, that implies a lower dependency between *β*-turn and *β*-sheets structural domain fractions. As a consequence, the structural transition analysis points out that the formation of antiparallel *β*-sheets domains relies on the availability of *α*-helix, *β*-turn monomers.

## Conclusion

Morphoscanner2.0 is a Python library for secondary structure analysis of protein/peptide structures in both all-atom and coarse-grained MD simulations. In a previous version, Morphoscanner recognized, tracked and provided insights of the development of protein *β*-structuration in MD simulations [18]. For the same reason, Morphoscanner has been successfully applied for classifying the failure mode of self-assembling peptides fibril seeds and fibrils [22]. On the other hand, Morphoscanner2.0 can now track the emergence of *α*-helix structural domains in protein structure and MD simulations. In particular, Morphoscanner2.0 has been used for the analysis of MARTINI CG-MD simulations of *α*-helix forming peptides [36, 50]. The analyses of these simulations elucidated the role of the different amino-acids on *α*-helix and *β*-sheet folding propensity. Further, this approach corroborated the use of MARTINI CG-MD simulations for studying peptide and protein folding [36, 51]. We then implemented a workflow for unveiling hidden mechanisms that promote self-assembling of BMHP1-derived SAPs [9, 57]. The workflow consisted of a combination of restrained MD simulations and analysis with Morphoscanner2.0 [53]. As shown in Fig 4, this workflow highlighted the mutual dependency of *β*-sheet and *α*-helix domains, then unveiling that the formation of antiparallel *β*-sheets domains relies on the availability of *α*-helix conformers more than *β*-turn monomers. Lastly, we developed a Python library useful for the analysis of peptide assembly, easily adaptable to other chemical species and coarsening levels, thanks to its interoperability with other Python libraries, such as MDAnalysis, MDtraj, Scipy, PyTorch, and Matplotlib [24–29] In summary, Morphoscanner2.0 is the only available software capable of a deep characterization of secondary structures development in coarse-grained MD simulations of peptides/proteins, but it can be potentially applied to the study of other biological processes such as DNA, RNA hybridization or abnormal protein assemblies [1, 31, 50].

## Supporting information

S1 File CG-MD simulation of *α*-helix forming peptides [36, 50, 56, 58–62]. (PDF)

S1 Fig Morphoscanner trajectory class structure description. The core object in Morphoscanner is the trajectory, denoted by **morphoscanner.trajectory.trajectory()**. The input required by Morphoscanner are highlighted in yellow. The methods of the **trajectory** are highlighted in light blue. The output of the different methods are highlighted in green. The **trajectory()** class need the file path of the initial configuration of the system and the file path of the trajectory file. The **explore()** method collects a series of data from the first frame of the trajectory. This method returns a list of **int** (**peptide_length_list**) where each entry is the number of amino acids of a peptide in the system. In addition, this method returns a dictionary (**len_dict**), that represents the distribution of the peptides with a certain length. The collected data for each peptide at the first frame are: amino acidic sequence (**sequence**), atom index (**atom_numbers**) and coordinates. The method **compose_database** parses the data from the first frame. The method **analyze_InLoop** takes three parameters, such as the contact threshold, the minimum number of contact for identifying a *β*-sheet structure, and the parallelization option (**device**). The analysis can be parallelized on CPU or GPU. The method **get_data()** run a set of other methods that recover data from the analysis. The obtained data are saved in a pandas dataframe.
(TIF)

S2 Fig Morphoscanner plotting functions. The method **plot_graph()** represents a graph that quantify the interactions between different protein or peptides, shown in S4 and S6 Figs. The method **plot_frame_aggregates()** is used for plotting a frame with a color code that identify the sense of the majority of contacts in a cluster, as shown in Fig 4B. The method **plot_contacts()** plots the ratio between antiparallel and total contact for each sampled frames, as shown in Fig 4C. The method **plot_peptides_in_beta()** plots the ratio between the number of peptides that form *β*-sheet structures and the total number of peptides, as shown in Fig 4D. The methods **plot3d_parallel()**, **plot3d_antiparallel_negative()**, **plot3d_antiparallel_positive()** and **gnuplot_shift_profile()** return the distribution of shift values overtime, as shown in Fig 5A–5C.
(TIF)

S3 Fig Secondary structure assignment and visualization using Morphoscanner and STRIDE output. A) The path of the location of the structure is assigned in string format. B) The **trajectory** object stores the information about the CG structure. C) A dedicated python script parses the plain text output (*txt file) from the web server STRIDE analysis. D,E) The lists of residues, that belong to *α*-helix domains, have been used for highlighting the *α*-helix domains in the atomistic structure.
(TIF)

S4 Fig Integration of Morphoscanner with MDAnalysis, NGLview and Networkx. A) Morphoscanner can be easily integrated into the same workflow with other libraries, such NGLview, Matplotlib, Numpy [28, 35, 63]. B) Morphoscanner, analogously to MDAnalysis, creates a class instance for the trajectories. C) The function *explore*() is needed to initialize a trajectory. The input MD trajectory can be analyzed on each frame by selecting the sampling interval. The analysis can be performed by selecting different distance threshold. The *get*_*data*() function is necessary for retrieving the results of the analysis. The *helix*_*score*() functions implements iteratively the algorithm 3. D) The results of the analysis can be used for plotting the structural domains using NGLview [63]. E) Morphoscanner2.0 leverages on Networkx [23] for plotting the contact graph of each frame.
(TIF)

S5 Fig Integration of Morphoscanner with PyInform. A) Morphoscanner can be easily integrated into the same workflow with other libraries, such NGLview, Matplotlib, Numpy, and a function can be defined for calculating the different statistics from each frame about *α*-helix structural domains [28, 35, 63]. B) A function can be defined for calculating the different statistics from each frame about *β*-sheet structural domains. In C),D) Morphoscanner analysis has been integrated in the same workflow with PyInform library functions for the calculation of mutual information and relative entropy [56]. The mutual information is calculated by considering the time-series of *α*-helix and *β*-sheet domains over time the time-series of structural domains are used to construct the empirical distributions of two random variables, so the mutual information can be computed.
(TIF)

S6 Fig Contact Graph visualization *α*-helix module. Morphoscanner plots the graph of one of the sampled frames with qualitative visual indications. The edge thickness is proportional to the number of contacts between two molecular subunits. The blue edges refer to antiparallel contacts, while green edges refer to parallel contact.
(TIF)

S7 Fig Tracking of *α*-helix structures in CG-MD simulations. A) Peptide M13 exhibits a great *α*-helix folding propensity, with a stability at 58.24% and the maximum *α*-helix content at 62.5% (See Table 1 for details). B)Peptide M17 shows a similar behavior to that of peptide M13 (See Table 1 for details). Indeed, the stability value is equal to 52.24% and the maximum *α*-helix content is 62.5% (See Table 1 for details). C)Peptide M34 shown a similar behavior to that of peptide M37 (See Fig 2 for details). D) M39 shares some features with peptide M40 but it is less stable. Indeed, the stability value is 32.46% and the maximum *α*-helix content is 62.5% (See Table 1 for comparison). E,F,G) Peptides M46, M47, M48 exhibit a lower propensity to fold in *α*-helix. Indeed, the average *α*-helix content is 11.71%, 8.65%, 11% for peptide M46, M47, M48 respectively.
(TIF)

S8 Fig Tracking of *β*-turn structures in CG-MD simulations. A) Peptide M13 is characterized by a low propensity to fold in *β*-turn structure. Indeed, the average *β*-turn content is equal to 3.40%, with a maximum peak equal to 37.5% In addition, the stability score is equal to 22.87%. (See Table 1 for details) B) Peptide M17 shows similar tendencies to peptide M13. Indeed, the average *β*-turn content is equal to 3.90%, with a maximum peak equal to 37.5% In addition, the stability score is equal to 25.77%. (See Table 1 for details) C) Peptide M34 is characterized by a low propensity to fold in *β*-turn structure. Indeed, the average *β*-turn content is equal to 2.23%, with a maximum peak equal to 37.5% and the stability score is equal to 15.28%. (See Table 1 for details) D) Peptide M39 is characterized by a low propensity to fold in *β*-turn structure. Indeed, the average *β*-turn content is equal to 4.10%, with a maximum peak equal to 37.5% and a stability score is equal to 27.57%. (See Table 1 for details) E,F,G) For eptides M46, M47, M48 similar conclusion can be drawn as shown in S7 Fig. Peptides M46, M47, M48 show a lower propensity to adopt a *β*-turn conformation, as demonstrated by their average *β*-turn content equal to 1.91%, 1.17%, 1.08% for peptide M46, M47, M48 respectively. (See Table 1 for details).
(TIF)
