# Supplementary material for: Morphoscanner2.0: A new python module for analysis of molecular dynamics simulations
Source: PLoS One. 2023 Apr 27;18(4):e0284307. doi: 10.1371/journal.pone.0284307 (PMC10138828; doi:10.1371/journal.pone.0284307)
Supplement: S1 File — (PDF) [file pone.0284307.s001.pdf]

Supporting information

CG-MD simulation of α-helix forming peptides The sequences of the simulated α-helix forming peptides are listed in Table 1. All-trans configuration of the peptides were generated by Pymol (http://www.pymol.org/). At neutral pH, lysine and glutamic acid, histidine side chains, because of their weak basic and acidic nature, can be considered fully protonated and deprotonated, respectively. Boxes, filled with MARTINI 2.0 CG water beads, ions (Na+ and Cl−) were added to neutralize the systems. The production phase was conducted using constant temperature, pressure, and number of molecules (i.e., the NPT ensemble). MD simulations were performed using the version 4.5.5 of GROMACS package [58, 59]. In MARTINI 2.2, coarse-grained molecular dynamics (CG-MD) simulations it is necessary to define peptide secondary structures, and the above mentioned parameters, to which individual amino acid residue must evolve [36]. The secondary structures have been assigned according to the results from Ho and Dill [50]. Prior to the production phase, the systems underwent to an equilibration phase (a 3000-steps minimization using steepest descents method) in order to eliminate high-energy interactions. The production phase was conducted in the NPT ensemble for 10 ns and the frames have been saved every 100 ps. Solutes and solvent were coupled independently to an external bath (T = 310 K) with a coupling constant (τT) of 1 ps using v-rescale thermostat [60]. Periodic boundary conditions were imposed, and pressure was maintained at 1 bar using the Berendsen coupling. The isothermal compressibility was set at 3 * 10-4 bar-1 and the coupling constant (τP) was 1 ps [61]. The constraints on lengths and angles of the bonds were applied with the LINCS algorithm [62].

Calculation of the mutual information The mutual information is a measure of the amount of mutual dependence between two random variables. As shown in Fig. S5, we calculated the mutual information between α-helix and β-sheet domains identified by Morphoscanner. Locally, the mutual information can be defined as follows:

$$m_i(\alpha, \beta) = -log_2 \frac{p(\beta, \alpha)}{p(\beta) * p(\alpha)} \tag{18}$$

Where, $p(\alpha)$ and $p(\beta)$ represent the percentage of α-helix and β-sheet domain calculated via Morphoscanner, respectively. In addition, $p(\beta, \alpha)$ represent the percentage of β-structures depending on α-structures. The mutual information is then calculated as the time average of $m_i(\alpha, \beta)$, as follows [56]:

$$M(\alpha, \beta) = -\sum_{\alpha_i, \beta_i} p(\beta, \alpha) log_2 \frac{p(\beta, \alpha)}{p(\beta) * p(\alpha)} \tag{19}$$
