# Supplementary figures and images for: Morphoscanner2.0: A new python module for analysis of molecular dynamics simulations
Source: PLoS One. 2023 Apr 27;18(4):e0284307. doi: 10.1371/journal.pone.0284307 (PMC10138828; doi:10.1371/journal.pone.0284307)

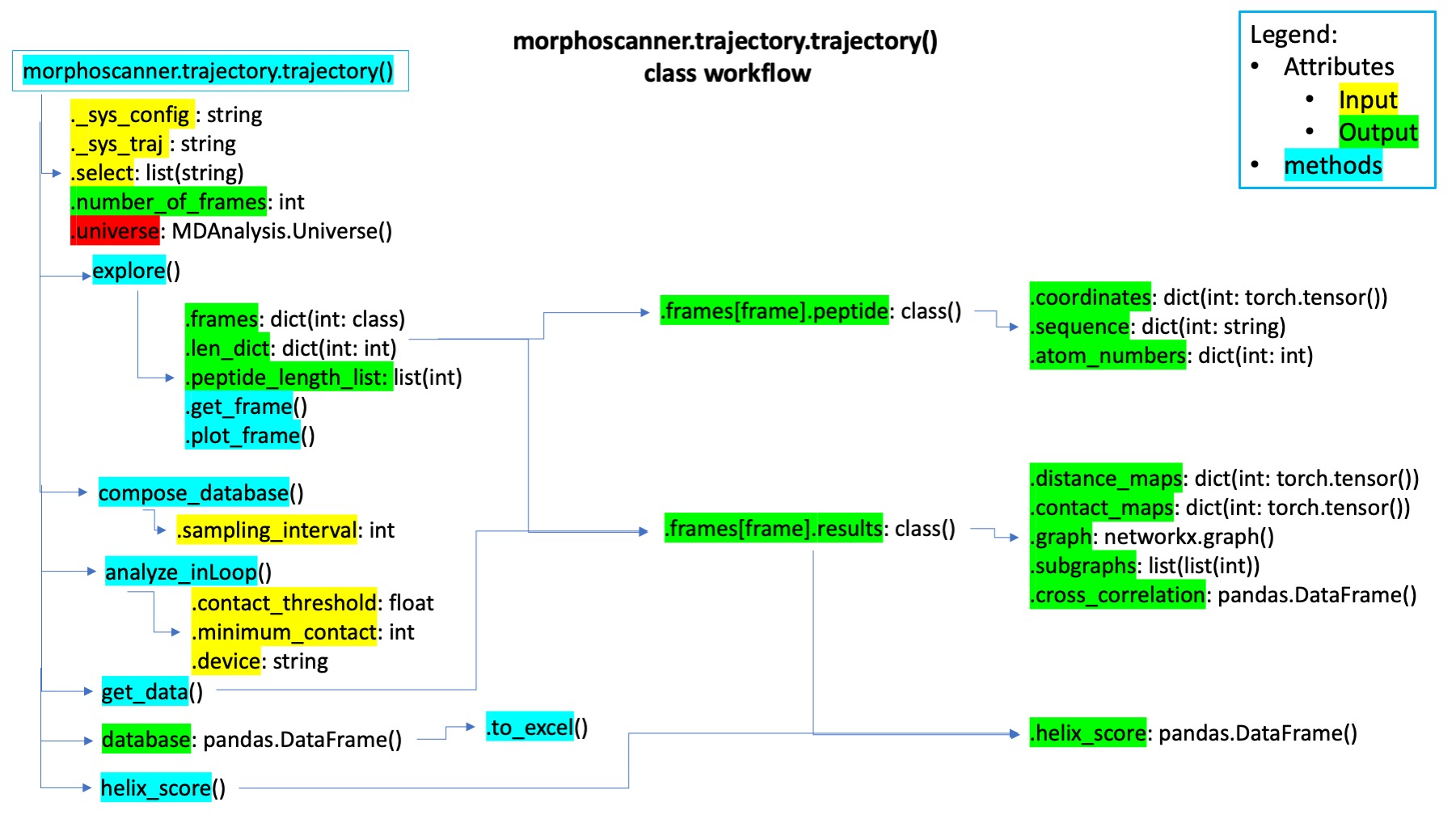

Supplement: S1 Fig — The core object in Morphoscanner is the trajectory, denoted by morphoscanner.trajectory.trajectory(). The input required by Morphoscanner are highlighted in yellow. The methods of the trajectory are highlighted in light blue. The output of the different methods are highlighted in green. The trajectory() class need the file path of the initial configuration of the system and the file path of the trajectory file. The explore() method collects a series of data from the first frame of the trajectory. This method returns a list of int (peptide_length_list) where each entry is the number of amino acids of a peptide in the system. In addition, this method returns a dictionary (len_dict), that represents the distribution of the peptides with a certain length. The collected data for each peptide at the first frame are: amino acidic sequence (sequence), atom index (atom_numbers) and coordinates. The method compose_database parses the data from the first frame. The method analyze_InLoop takes three parameters, such as the contact threshold, the minimum number of contact for identifying a β-sheet structure, and the parallelization option (device). The analysis can be parallelized on CPU or GPU. The method get_data() run a set of other methods that recover data from the analysis. The obtained data are saved in a pandas dataframe. (TIF) [file pone.0284307.s002.tif]

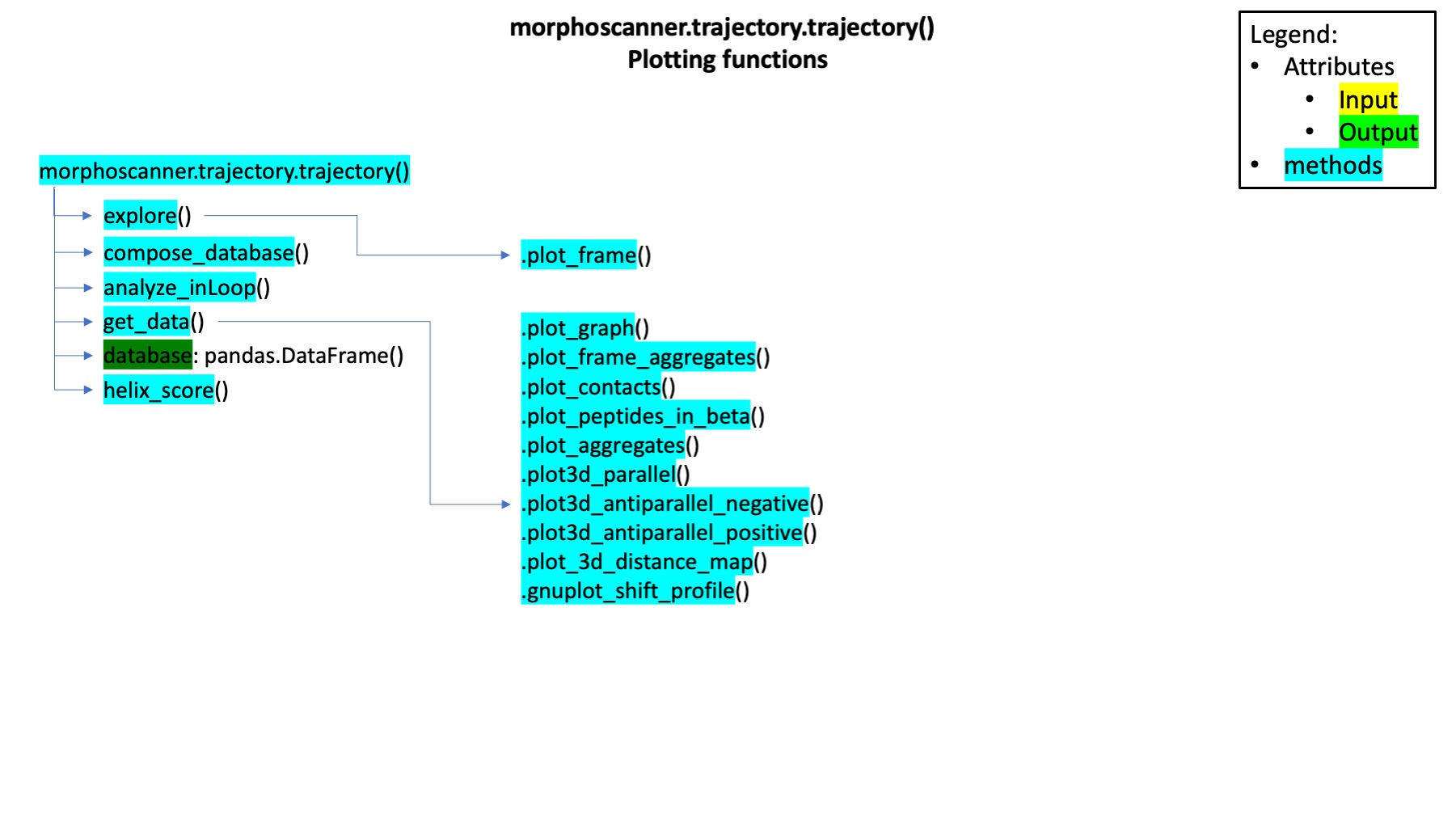

Supplement: S2 Fig — The method plot_graph() represents a graph that quantify the interactions between different protein or peptides, shown in S4 and S6 Figs. The method plot_frame_aggregates() is used for plotting a frame with a color code that identify the sense of the majority of contacts in a cluster, as shown in Fig 4B. The method plot_contacts() plots the ratio between antiparallel and total contact for each sampled frames, as shown in Fig 4C. The method plot_peptides_in_beta() plots the ratio between the number of peptides that form β-sheet structures and the total number of peptides, as shown in Fig 4D. The methods plot3d_parallel(), plot3d_antiparallel_negative(), plot3d_antiparallel_positive() and gnuplot_shift_profile() return the distribution of shift values overtime, as shown in Fig 5A–5C. (TIF) [file pone.0284307.s003.tif]

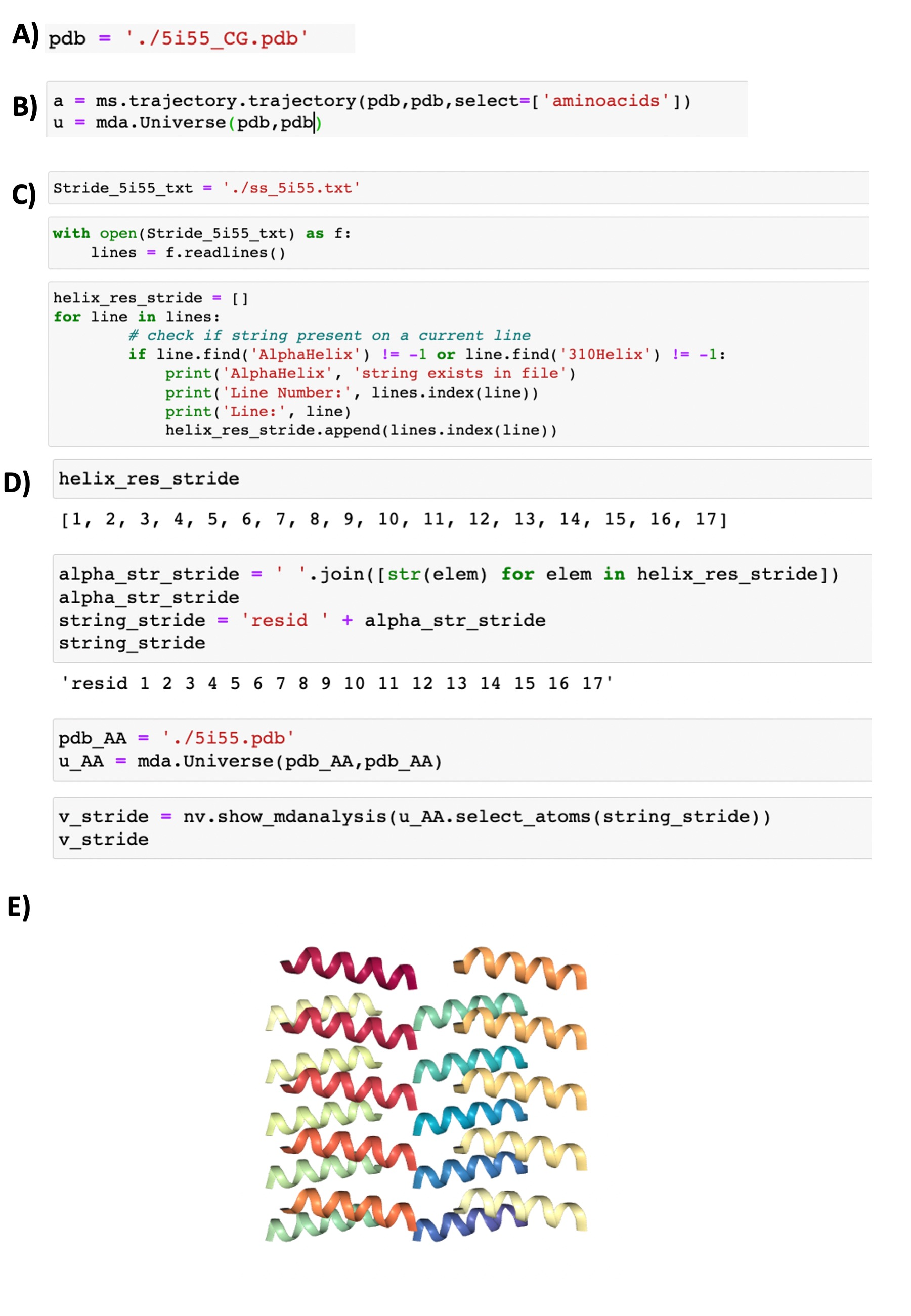

Supplement: S3 Fig — A) The path of the location of the structure is assigned in string format. B) The trajectory object stores the information about the CG structure. C) A dedicated python script parses the plain text output (*txt file) from the web server STRIDE analysis. D,E) The lists of residues, that belong to α-helix domains, have been used for highlighting the α-helix domains in the atomistic structure. (TIF) [file pone.0284307.s004.tif]

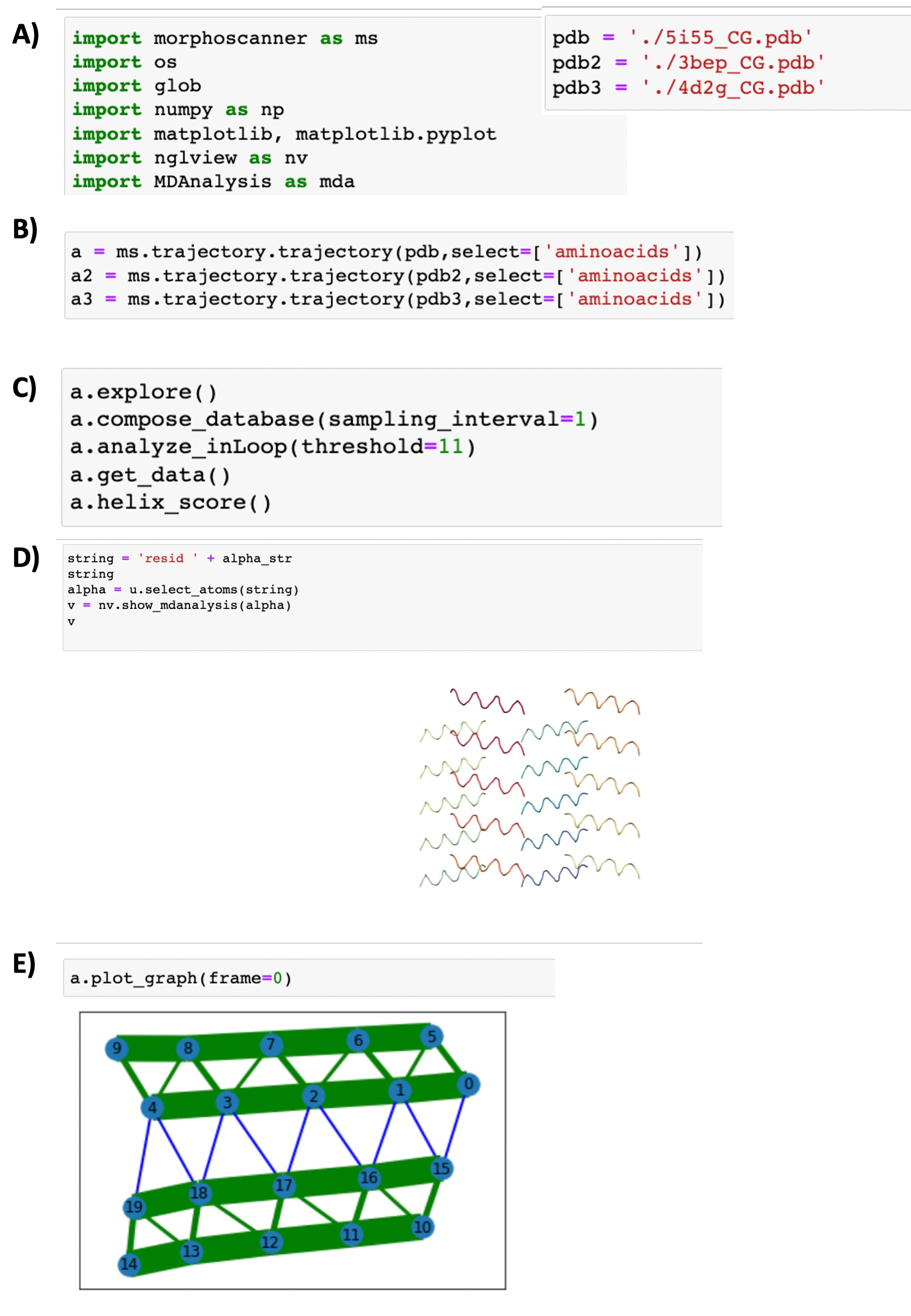

Supplement: S4 Fig — A) Morphoscanner can be easily integrated into the same workflow with other libraries, such NGLview, Matplotlib, Numpy [28, 35, 63]. B) Morphoscanner, analogously to MDAnalysis, creates a class instance for the trajectories. C) The function explore() is needed to initialize a trajectory. The input MD trajectory can be analyzed on each frame by selecting the sampling interval. The analysis can be performed by selecting different distance threshold. The get_data() function is necessary for retrieving the results of the analysis. The helix_score() functions implements iteratively the algorithm 3. D) The results of the analysis can be used for plotting the structural domains using NGLview [63]. E) Morphoscanner2.0 leverages on Networkx [23] for plotting the contact graph of each frame. (TIF) [file pone.0284307.s005.tif]

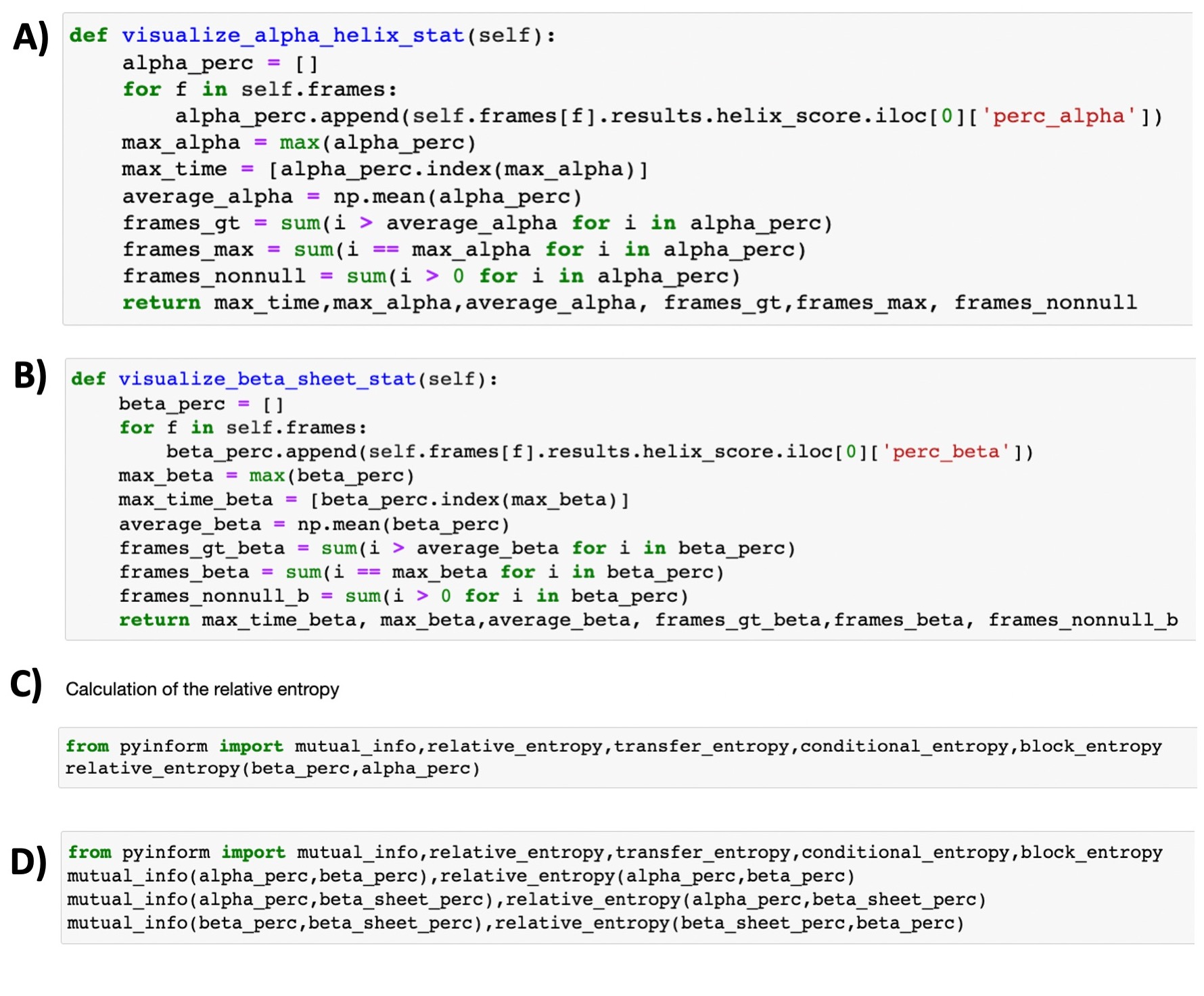

Supplement: S5 Fig — A) Morphoscanner can be easily integrated into the same workflow with other libraries, such NGLview, Matplotlib, Numpy, and a function can be defined for calculating the different statistics from each frame about α-helix structural domains [28, 35, 63]. B) A function can be defined for calculating the different statistics from each frame about β-sheet structural domains. In C),D) Morphoscanner analysis has been integrated in the same workflow with PyInform library functions for the calculation of mutual information and relative entropy [56]. The mutual information is calculated by considering the time-series of α-helix and β-sheet domains over time the time-series of structural domains are used to construct the empirical distributions of two random variables, so the mutual information can be computed. (TIF) [file pone.0284307.s006.tif]

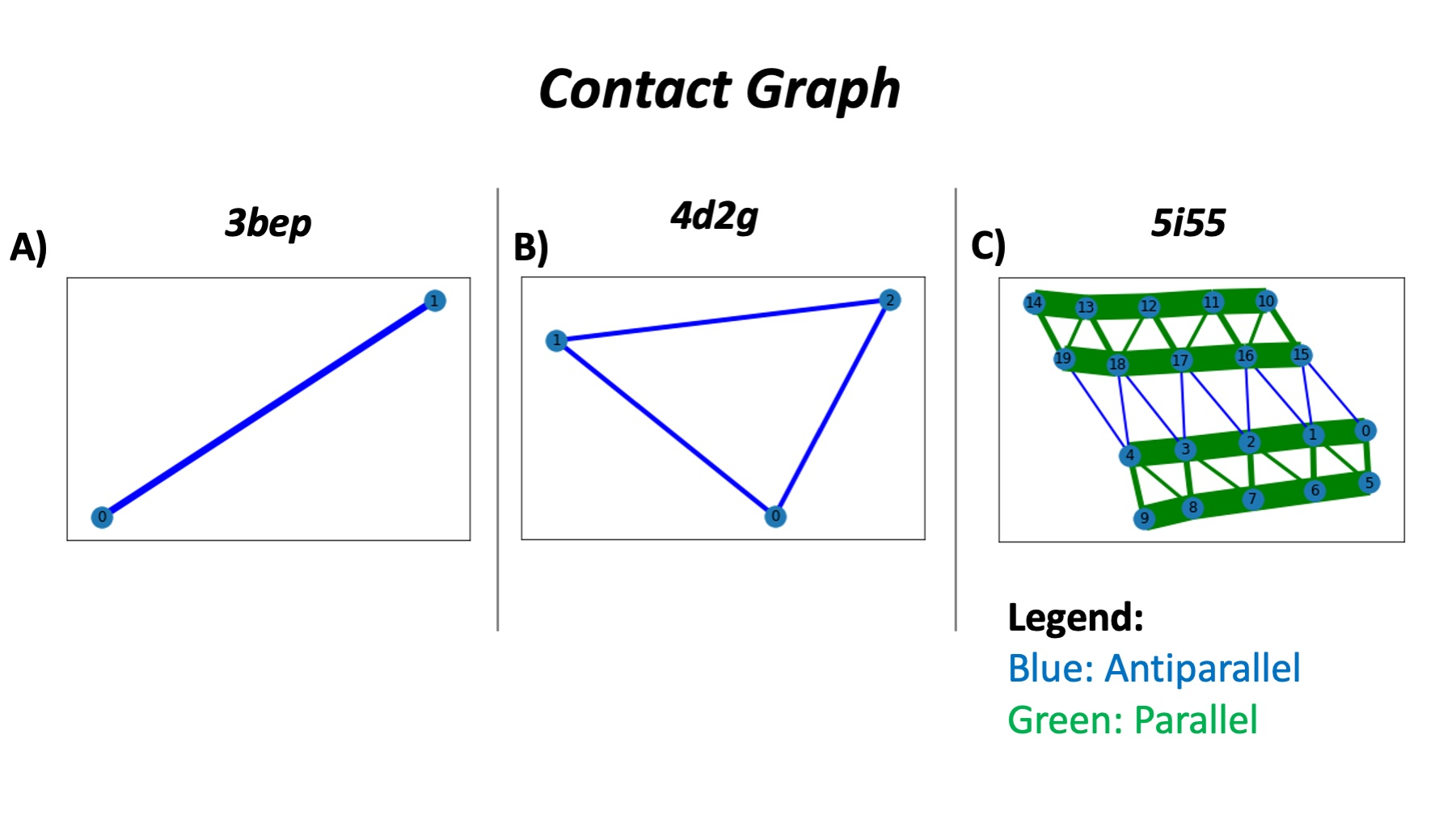

Supplement: S6 Fig — Morphoscanner plots the graph of one of the sampled frames with qualitative visual indications. The edge thickness is proportional to the number of contacts between two molecular subunits. The blue edges refer to antiparallel contacts, while green edges refer to parallel contact. (TIF) [file pone.0284307.s007.tif]

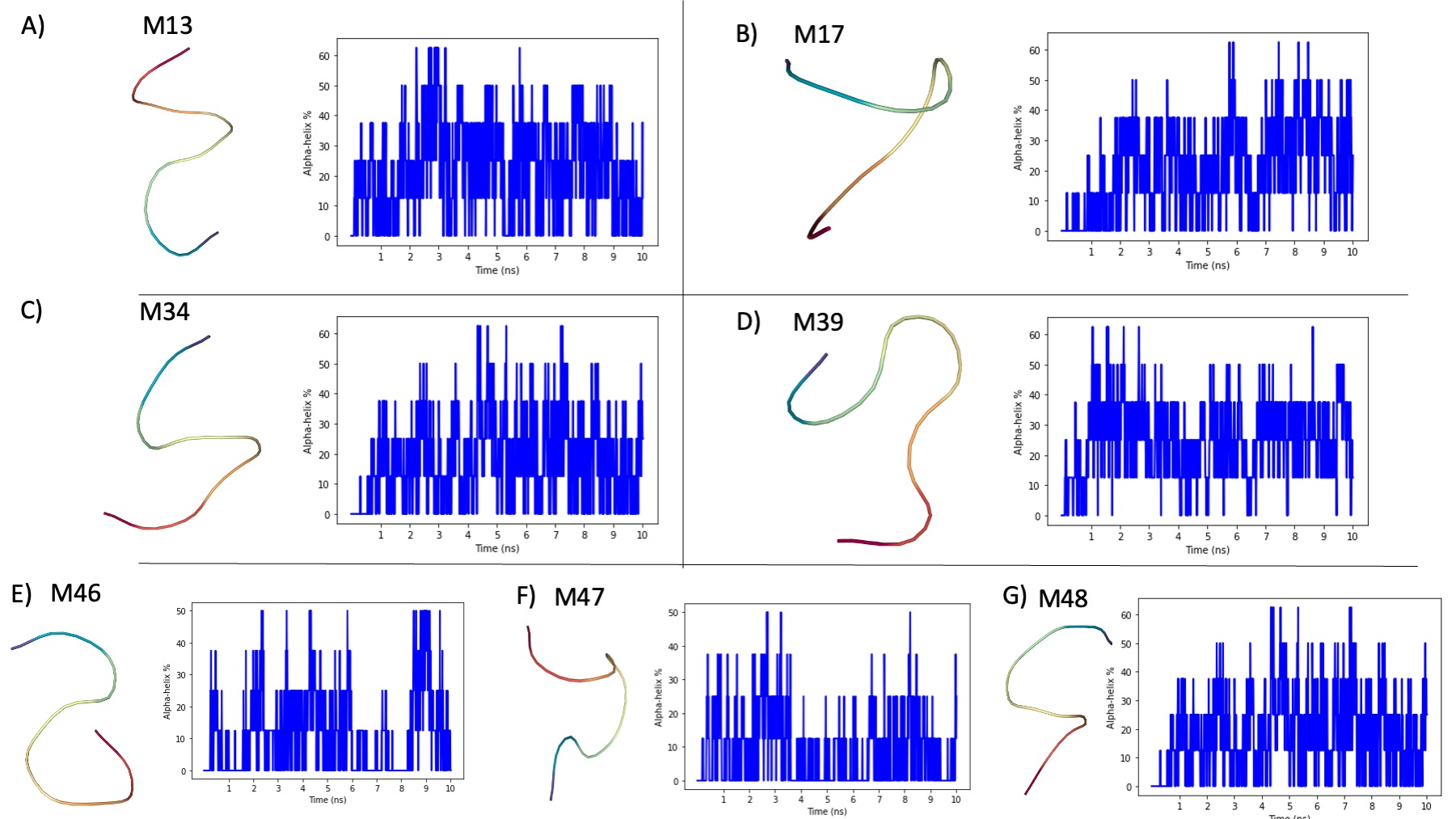

Supplement: S7 Fig — A) Peptide M13 exhibits a great α-helix folding propensity, with a stability at 58.24% and the maximum α-helix content at 62.5% (See Table 1 for details). B)Peptide M17 shows a similar behavior to that of peptide M13 (See Table 1 for details). Indeed, the stability value is equal to 52.24% and the maximum α-helix content is 62.5% (See Table 1 for details). C)Peptide M34 shown a similar behavior to that of peptide M37 (See Fig 2 for details). D) M39 shares some features with peptide M40 but it is less stable. Indeed, the stability value is 32.46% and the maximum α-helix content is 62.5% (See Table 1 for comparison). E,F,G) Peptides M46, M47, M48 exhibit a lower propensity to fold in α-helix. Indeed, the average α-helix content is 11.71%, 8.65%, 11% for peptide M46, M47, M48 respectively. (TIF) [file pone.0284307.s008.tif]

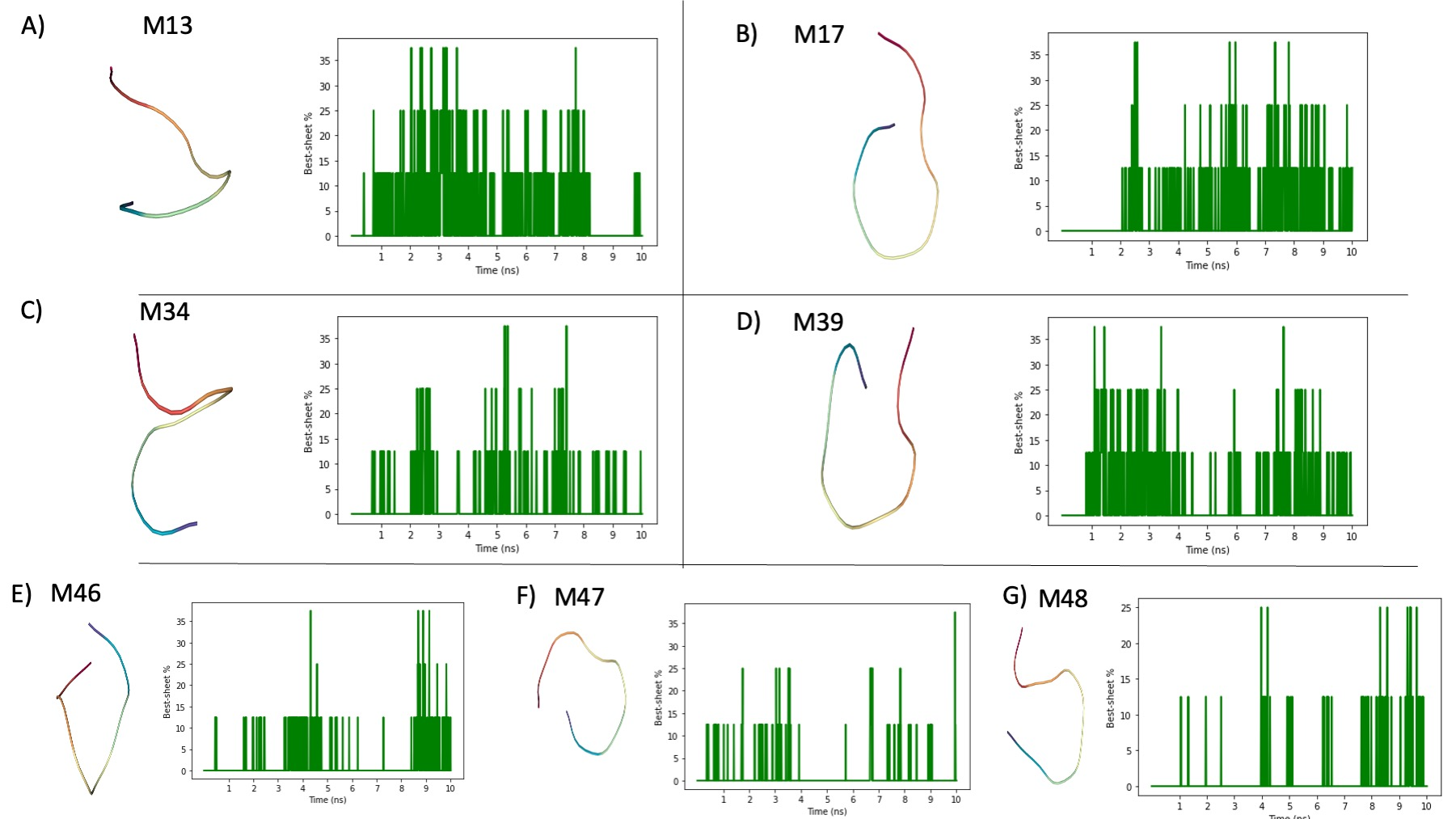

Supplement: S8 Fig — A) Peptide M13 is characterized by a low propensity to fold in β-turn structure. Indeed, the average β-turn content is equal to 3.40%, with a maximum peak equal to 37.5% In addition, the stability score is equal to 22.87%. (See Table 1 for details) B) Peptide M17 shows similar tendencies to peptide M13. Indeed, the average β-turn content is equal to 3.90%, with a maximum peak equal to 37.5% In addition, the stability score is equal to 25.77%. (See Table 1 for details) C) Peptide M34 is characterized by a low propensity to fold in β-turn structure. Indeed, the average β-turn content is equal to 2.23%, with a maximum peak equal to 37.5% and the stability score is equal to 15.28%. (See Table 1 for details) D) Peptide M39 is characterized by a low propensity to fold in β-turn structure. Indeed, the average β-turn content is equal to 4.10%, with a maximum peak equal to 37.5% and a stability score is equal to 27.57%. (See Table 1 for details) E,F,G) For eptides M46, M47, M48 similar conclusion can be drawn as shown in S7 Fig. Peptides M46, M47, M48 show a lower propensity to adopt a β-turn conformation, as demonstrated by their average β-turn content equal to 1.91%, 1.17%, 1.08% for peptide M46, M47, M48 respectively. (See Table 1 for details). (TIF) [file pone.0284307.s009.tif]
